# Inertial proximal alternating minimization for nonconvex and nonsmooth problems

**DOI:** 10.1186/s13660-017-1504-y

**Published:** 2017-09-20

**Authors:** Yaxuan Zhang, Songnian He

**Affiliations:** College of Science, Civil Aviation University of China, Tianjin, 300300 China

**Keywords:** nonconvex nonsmooth optimization, proximal alternating minimization, inertial, Kurdyka-Lojasiewicz inequality, convergence

## Abstract

In this paper, we study the minimization problem of the type $L(x,y)=f(x)+R(x,y)+g(y)$, where *f* and *g* are both nonconvex nonsmooth functions, and *R* is a smooth function we can choose. We present a proximal alternating minimization algorithm with inertial effect. We obtain the convergence by constructing a key function *H* that guarantees a sufficient decrease property of the iterates. In fact, we prove that if *H* satisfies the Kurdyka-Lojasiewicz inequality, then every bounded sequence generated by the algorithm converges strongly to a critical point of *L*.

## Introduction

Nonconvex and nonsmooth optimization problems are extremely useful in many applied sciences, including statistics, machine learning, regression, classification, and so on. One of the most practical and classical optimization problems is of the form

1 $$ \min_{x\in\mathbb {R}^{n}} \bigl[f(x)+g(x) \bigr]. $$

In this paper, we study the problem in the nonconvex and nonsmooth setting, where $f, g: \mathbb {R}^{n}\to(-\infty,\infty]$ are proper lower semicontinuous functions. We aim at finding the critical points of

2 $$ L(x,y)=f(x)+R(x,y)+g(y) $$

(with *R* being smooth) and possibly solving the corresponding minimization problem (1). This can be seen by setting

$$L(x,y)=f(x)+\frac{\rho}{2} \Vert x-y \Vert ^{2}+g(y), $$

where $\rho>0$ is a relaxation parameter.

For problem (2), we introduce a proximal alternating minimization algorithm with inertial effect and investigate the convergence of the generated iterates. Inertial proximal methods go back to [1, 2], where it has been noticed that the discretization of a differential system of second order in time gives rise to a generalization of the classical proximal-point algorithm. The main feature of the inertial proximal algorithm is that the next iterate is defined by using the last two iterates. Recently, there has been an increasing interest in algorithms with inertial effect; see [3–12].

Generally, we consider the problem

$$\min L(x,y)=\min \bigl\{ f(x)+R(x,y)+g(y) \bigr\} $$

with $x\in\mathbb {R}^{n}$ and $y\in\mathbb {R}^{m}$.

In [13], the authors proposed the alternating minimization algorithm

$$\textstyle\begin{cases} x_{k+1}\in\operatorname{argmin} \{L(u,y_{k})+\frac{1}{2\lambda _{k}} \Vert u-x_{k} \Vert ^{2}: u\in\mathbb {R}^{n} \},\\ y_{k+1}\in\operatorname{argmin} \{L(x_{k+1},v)+\frac{1}{2\mu _{k}} \Vert v-y_{k} \Vert ^{2}: v\in\mathbb {R}^{m} \}, \end{cases} $$

which can be viewed as a proximal regularization of a two-block Gauss-Seidel method for minimizing *L*,

$$\textstyle\begin{cases} x_{k+1}\in\operatorname{argmin} \{L(u,y_{k}): u\in\mathbb {R}^{n} \},\\ y_{k+1}\in\operatorname{argmin} \{L(x_{k+1},v): v\in\mathbb {R}^{m} \}. \end{cases} $$

Inspired by [13], we propose the algorithm

3 $$\begin{aligned} & x_{k+1}\in \mathop{\operatorname{argmin}}_{x\in\mathbb {R}^{n}} \biggl\{ L(x,y_{k})+\frac {1}{2\lambda_{k}} \Vert x-x_{k} \Vert ^{2}+\alpha_{k}\langle x,x_{k}-x_{k-1} \rangle \biggr\} , \end{aligned}$$

4 $$\begin{aligned} & y_{k+1}\in \mathop{\operatorname{argmin}}_{y\in\mathbb {R}^{m}} \biggl\{ L(x_{k+1},y)+\frac {1}{2\mu_{k}} \Vert y-y_{k} \Vert ^{2}+\beta_{k}\langle y,y_{k}-y_{k-1} \rangle \biggr\} . \end{aligned}$$

We need the following assumptions on the functions and parameters.

(H1): $f: \mathbb {R}^{n}\to(-\infty,\infty]$ and $g: \mathbb {R}^{m}\to (-\infty,\infty]$ are proper lower semicontinuous functions;
(H2): $R: \mathbb {R}^{n}\times\mathbb {R}^{m}\to\mathbb {R}$ is a continuously differentiable function;
(H3): ∇*R* is Lipschitz continuous on bounded subsets of $\mathbb {R}^{n}\times\mathbb {R}^{m}$;
(H4): $\inf L>-\infty$;
(H5): $0<\mu_{-}\leq\mu_{k}\leq\mu_{+}, 0<\lambda_{-}\leq\lambda_{k}\leq \lambda_{+}, 0\leq\alpha_{k}\leq\alpha, 0\leq\beta_{k}\leq\beta$;
(H6): $\sigma>\max\{\alpha,\beta\}\cdot\max\{\lambda_{+},\mu_{+}\} \cdot(\sigma^{2}+1)$.

To prove the convergence of the algorithm under these assumptions, we construct a key function *H*, which is defined as in (11). Based on *H*, we can obtain a sufficient decrease property of the iterates, the existence of a subgradient lower bound for the iterate gap, and some important analytic features of the objective function. Finally, we can prove that every bounded sequence generated by the algorithm converges to a critical point of *L* if *H* satisfies the Kurdyka-Lojasiewicz inequality.

The rest of the paper is arranged as follows. In Section 2, we recall some elementary notions and facts of nonsmooth nonconvex analysis. In Section 3, we present a detailed proof of the convergence of the algorithm. In Section 4, we give a brief conclusion.

## Preliminaries

In this section, we recall some definitions and results. Let $\mathbb {N}$ be the set of nonnegative integers. For $m\geq1$, the Euclidean scalar product and induced norm on $\mathbb {R}^{m}$ are denoted by $\langle\cdot ,\cdot\rangle$ and $\Vert \cdot \Vert $, respectively.

The *domain* of a function $f:\mathbb {R}^{m}\to(-\infty,\infty]$ is defined by $\operatorname{dom} f=\{x\in\mathbb {R}^{m}: f(x)<\infty\}$. We say that *f* is *proper* if $\operatorname{dom} f\neq\emptyset$. For the following generalized subdifferential notions and their basic properties we refer to [14] and [15]. Let $f:\mathbb {R}^{m}\to(-\infty,\infty]$ be a proper lower semicontinuous function. For $x\in\operatorname{dom} f$, we consider the *Frechet (viscosity) subdifferential* of *f* at *x* defined by the set

5 $$ \hat{\partial}f(x)= \biggl\{ v\in\mathbb {R}^{m}: \liminf _{y\to x}\frac {f(y)-f(x)-\langle v,y-x\rangle }{ \Vert y-x \Vert }\geq0 \biggr\} . $$

For $x\notin\operatorname{dom} f$, we set $\hat{\partial}f(x):=\emptyset$. The *limiting (Mordukhovich) subdifferential* *of* *f* at $x\in \operatorname{dom} f$ is defined by

6 $$ \partial f(x)= \bigl\{ v\in\mathbb {R}^{m}: \exists x_{n}\to x, f(x_{n})\to f(x) \text{ and } \exists v_{n}\in\hat{\partial}f(x_{n}), v_{n}\to v \text{ as } n\to\infty \bigr\} , $$

whereas for $x\notin\operatorname{dom} f$, we take $\partial f(x):=\emptyset$.

It is known that both notions of subdifferentials coincide with the *convex subdifferential* if *f* is convex, that is, $\hat{\partial}f(x)=\partial f(x)=\{v\in\mathbb {R}^{m}:f(y)\geq f(x)+\langle v,y-x\rangle, \forall y\in\mathbb {R}^{m}\}$. Notice that if *f* is continuously differentiable around $x\in\mathbb {R}^{m}$, then we have $\partial f(x)=\{\nabla f(x)\}$. Generally, the inclusion $\hat{\partial}f(x)\subset\partial f(x)$ holds for each $x\in\mathbb {R}^{m}$.

The *Fermat rule* reads in this nonsmooth setting as follows: if $x\in\mathbb {R}^{m}$ is a local minimizer of *f*, then

$$0\in\partial f(x). $$

Denote by

$$\operatorname{crit} f= \bigl\{ x\in\mathbb {R}^{m}: 0\in\partial f(x) \bigr\} $$

the set of *(limiting) critical points* of *f*. Let us mention also the following subdifferential rule: if $f: \mathbb {R}^{m}\to(-\infty ,\infty]$ is proper lower semicontinuous and $g: \mathbb {R}^{m}\to \mathbb {R}$ is a continuously differentiable function, then

$$\partial(f+g) (x)=\partial f(x)+\nabla g(x) $$

for all $x\in\mathbb {R}^{m}$.

We also denote $\operatorname{dist}(x,\Omega)=\inf_{y\in\Omega} \Vert x-y \Vert $ for $x\in\mathbb {R}^{m}$ and $\Omega\subset\mathbb {R}^{m}$.

Now let us recall the *Kurdyka-Lojasiexicz property*, which plays an important role in the proof of the convergence of our algorithm.

### Definition 2.1

Kurdyka-Lojasiexicz property; see [13, 16]

Let $f: \mathbb {R}^{m}\to(-\infty,\infty]$ be a proper lower semicontinuous function. We say that *f* satisfies the *Kurdyka-Lojasiexicz (KL) property* at $\bar{x}\in\operatorname{dom} \partial f=\{ x\in\mathbb {R}^{m}: \partial f(x)\neq\emptyset\}$ if there exist $\eta >0$, a neighborhood *U* of *x̄*, and a continuous concave function $\varphi: [0,\eta)\to[0,\infty)$ such that

(i) $\varphi(0)=0$;
(ii) *φ* is continuously differentiable on $(0,\eta)$ and continuous at 0;
(iii) $\varphi'(s)>0$ for all $s\in(0,\eta)$;
(iv) for all $x\in U\cap\{x\in\mathbb {R}^{m}: f(\bar{x})< f(x)< f(\bar{x})+\eta\}$, we have the *KL inequality*:
  7 $$ \varphi' \bigl(f(x)-f(\bar{x}) \bigr) \operatorname{dist} \bigl(0, \partial f(x) \bigr)\geq1. $$
  If *f* satisfies the KL property at each point in $\operatorname{dom} \partial f$, then we call *f* a *KL function*.

It is worth mentioning that many functions in applied science are the KL functions (see [16]). In fact, semialgebraic functions, real subanalytic functions, semiconvex functions, and uniformly convex functions are all KL functions.

The following result (see [16], Lemma 6) is crucial to our convergence analysis.

### Lemma 2.1

*Let* $\Omega\subset\mathbb {R}^{m}$ *be a compact set*, *and let* $f: \mathbb {R}^{m}\to(-\infty,\infty]$ *be a proper lower semicontinuous function*. *Assume that* *f* *is constant on* Ω *and* *f* *satisfies the KL property at each point of* Ω. *Then there exist* $\epsilon>0, \eta>0$, *and a continuous concave function* *φ* *such that*

(i) $\varphi(0)=0$;
(ii) *φ* *is continuously differentiable on* $(0,\eta)$ *and continuous at* 0;
(iii) $\varphi'(s)>0$ *for all* $s\in(0,\eta)$;
(iv) *for all* $\bar{x}\in\Omega$ *and all* $x\in\{x\in\mathbb {R}^{m}: \operatorname{dist}(x,\Omega)<\epsilon\}\cap\{x\in\mathbb {R}^{m}: f(\bar{x})<f(x)<f(\bar{x})+\eta\}$, *we have the KL inequality*:
  8 $$ \varphi' \bigl(f(x)-f(\bar{x}) \bigr) \operatorname{dist} \bigl(0, \partial f(x) \bigr)\geq1. $$

We need the following two lemmas. The first one was often used in the context of Fejer monotonicity techniques for proving convergence results of classical algorithms for convex optimization problems or, more generally, for monotone inclusion problems (see [17]). The second one is easy to verify (see [12]).

### Lemma 2.2

*Let* $\{a_{n}\}_{n\in\mathbb {N}}$ *and* $\{b_{n}\}_{n\in\mathbb {N}}$ *be real sequences such that* $b_{n}\geq0$ *for all* $n\in\mathbb {N}$, $\{a_{n}\} _{n\in\mathbb {N}}$ *is bounded below*, *and* $a_{n+1}+b_{n}\leq a_{n}$ *for all* $n\in\mathbb {N}$. *Then* $\{a_{n}\}_{n\in\mathbb {N}}$ *is a monotonically decreasing and convergent sequence*, *and* $\sum_{n\in\mathbb {N}}b_{n}<+\infty$.

### Lemma 2.3

*Let* $\{a_{n}\}_{n\in\mathbb {N}}$ *and* $\{b_{n}\}_{n\in\mathbb {N}}$ *be nonnegative real sequences such that* $\sum_{n\in\mathbb {N}} b_{n}<\infty$ *and* $a_{n+1}\leq a\cdot a_{n}+b\cdot a_{n-1}+b_{n}$ *for all* $n\geq1$, *where* $a\in\mathbb {R}, b\geq0$, *and* $a+b<1$. *Then* $\sum_{n\in\mathbb {N}} a_{n}<\infty$.

## The convergence of the algorithm

In this section, we prove the convergence of our algorithm. Motivated by [11] and [13], we divide the proof into three main steps, which are listed in the following three subsections, respectively.

We always use $\{(x_{k},y_{k})\}$ as the sequence generated by (3)-(4).

### A sufficient decrease property of the iterates

In this subsection, we construct the key function *H* and prove that the iterates have a sufficient decrease property.

#### Lemma 3.1

*Under assumptions* (H1)-(H6), *the sequence* $\{(x_{k},y_{k})\}$ *is well defined*, *and* $\{L(x_{k},y_{k})\}$ *is decreasing*. *More precisely*, *there exist two positive constants* $m_{2}>m_{1}>0$ *such that*

9 $$\begin{aligned} & L(x_{k+1},y_{k+1})+m_{2} \bigl( \Vert x_{k+1}-x_{k} \Vert ^{2}+ \Vert y_{k+1}-y_{k} \Vert ^{2} \bigr) \\ &\quad\leq L(x_{k},y_{k})+m_{1} \bigl( \Vert x_{k}-x_{k-1} \Vert ^{2}+ \Vert y_{k}-y_{k-1} \Vert ^{2} \bigr). \end{aligned}$$

#### Proof

Assumption (H4) indicates that, for any $r_{1}, r_{2}>0$ and $(\bar{x},\bar{y}), (\hat{x},\hat{y})\in\mathbb {R}^{n}\times \mathbb {R}^{m}$, the functions

$$x\to L(x,\bar{y})+\frac{1}{2r_{1}} \Vert x-\bar{x} \Vert ^{2}+r_{2} \langle x,\bar{x}-\hat{x}\rangle $$

and

$$y\to L(\bar{x},y)+\frac{1}{2r_{1}} \Vert y-\bar{y} \Vert ^{2}+r_{2} \langle y,\bar{y}-\hat{y}\rangle $$

are coercive. Thus the sequence $\{(x_{k},y_{k})\}$ is well defined.

Now we prove (9). Using the definition of $x_{k+1}$ and $y_{k+1}$ in (3) and (4), we have

$$\begin{aligned} &L(x_{k+1},y_{k+1})+\frac{1}{2\mu_{k}} \Vert y_{k+1}-y_{k} \Vert ^{2}+\beta _{k} \langle y_{k+1},y_{k}-y_{k-1}\rangle \\ &\quad\leq L(x_{k+1},y_{k})+\beta_{k}\langle y_{k},y_{k}-y_{k-1}\rangle \\ &\quad =L(x_{k+1},y_{k})+\frac{1}{2\lambda_{k}} \Vert x_{k+1}-x_{k} \Vert ^{2}+\alpha _{k} \langle x_{k+1},x_{k}-x_{k-1}\rangle + \beta_{k}\langle y_{k},y_{k}-y_{k-1}\rangle \\ &\qquad{}-\frac{1}{2\lambda_{k}} \Vert x_{k+1}-x_{k} \Vert ^{2}-\alpha_{k}\langle x_{k+1},x_{k}-x_{k-1} \rangle \\ &\quad\leq L(x_{k},y_{k})+\alpha_{k}\langle x_{k},x_{k}-x_{k-1}\rangle +\beta _{k} \langle y_{k},y_{k}-y_{k-1}\rangle \\ &\qquad{}-\frac{1}{2\lambda_{k}} \Vert x_{k+1}-x_{k} \Vert ^{2}-\alpha_{k}\langle x_{k+1},x_{k}-x_{k-1} \rangle. \end{aligned}$$

This leads to

$$\begin{aligned} &L(x_{k+1},y_{k+1})+\frac{1}{2\lambda_{k}} \Vert x_{k+1}-x_{k} \Vert ^{2}+\frac {1}{2\mu_{k}} \Vert y_{k+1}-y_{k} \Vert ^{2} \\ &\quad\leq L(x_{k},y_{k})+\alpha_{k}\langle x_{k}-x_{k+1},x_{k}-x_{k-1}\rangle + \beta_{k}\langle y_{k}-y_{k+1},y_{k}-y_{k-1} \rangle \\ &\quad\leq L(x_{k},y_{k})+\alpha_{k} \Vert x_{k}-x_{k+1} \Vert \Vert x_{k}-x_{k-1} \Vert +\beta _{k} \Vert y_{k}-y_{k+1} \Vert \Vert y_{k}-y_{k-1} \Vert \\ &\quad \leq L(x_{k},y_{k})+\frac{\sigma\alpha_{k}}{2} \Vert x_{k}-x_{k+1} \Vert ^{2}+\frac {\alpha_{k}}{2\sigma} \Vert x_{k}-x_{k-1} \Vert ^{2} + \frac{\sigma\beta_{k}}{2} \Vert y_{k}-y_{k+1} \Vert ^{2}\\ &\qquad{}+\frac{\beta_{k}}{2\sigma } \Vert y_{k}-y_{k-1} \Vert ^{2} \end{aligned}$$

for any $\sigma>0$. Thus it yields

$$\begin{aligned} &L(x_{k+1},y_{k+1})+\frac{1}{2} \biggl( \frac{1}{\lambda_{k}}-\sigma\alpha _{k} \biggr) \Vert x_{k+1}-x_{k} \Vert ^{2}+\frac{1}{2} \biggl(\frac{1}{\mu_{k}}-\sigma\beta _{k} \biggr) \Vert y_{k+1}-y_{k} \Vert ^{2} \\ &\quad\leq L(x_{k},y_{k})+\frac{1}{2}\frac{\alpha_{k}}{\sigma } \Vert x_{k}-x_{k-1} \Vert ^{2}+\frac{1}{2} \frac{\beta_{k}}{\sigma} \Vert y_{k}-y_{k-1} \Vert ^{2}. \end{aligned}$$

Clearly assumption (H5) implies that

$$\frac{\alpha_{k}}{\sigma}\leq\frac{\alpha}{\sigma}:=2M_{1}, \qquad \frac {1}{\lambda_{k}}- \sigma\alpha_{k}\geq\frac{1}{\lambda_{+}}-\sigma \alpha:=2M_{3} $$

and

$$\frac{\beta_{k}}{\sigma}\leq\frac{\beta}{\sigma}:=2M_{2},\qquad \frac {1}{\mu_{k}}- \sigma\beta_{k}\geq\frac{1}{\mu_{+}}-\sigma\beta:=2M_{4}. $$

Thus

$$\begin{aligned} &L(x_{k+1},y_{k+1})+M_{3} \Vert x_{k+1}-x_{k} \Vert ^{2}+M_{4} \Vert y_{k+1}-y_{k} \Vert ^{2}\\ &\quad\leq L(x_{k},y_{k})+M_{1} \Vert x_{k}-x_{k-1} \Vert ^{2}+M_{2} \Vert y_{k}-y_{k-1} \Vert ^{2}. \end{aligned}$$

Set $m_{1}=\max\{M_{1}, M_{2}\}, m_{2}=\min\{M_{3},M_{4}\}$. Then

10 $$\begin{aligned} & L(x_{k+1},y_{k+1})+m_{2} \bigl( \Vert x_{k+1}-x_{k} \Vert ^{2}+ \Vert y_{k+1}-y_{k} \Vert ^{2} \bigr) \\ &\quad\leq L(x_{k},y_{k})+m_{1} \bigl( \Vert x_{k}-x_{k-1} \Vert ^{2}+ \Vert y_{k}-y_{k-1} \Vert ^{2} \bigr). \end{aligned}$$

An elementary verification shows that $m_{2}>m_{1}>0$ under assumption (H6). □

#### Remark 3.1

Based on Lemma 3.1, we can define the new function

11 $$ H(z,w)=L(x,y)+m_{1} \Vert z-w \Vert ^{2}, $$

where $z=(x,y), w=(u,v)$, and $\Vert z-w \Vert ^{2}= \Vert x-u \Vert ^{2}+ \Vert y-v \Vert ^{2}$. Set $z_{k}=(x_{k},y_{k})$. Then Lemma 3.1 implies that the sequence $\{H(z_{k+1},z_{k})\}$ is decreasing. The decrease property of the iterates $\{x_{k},y_{k}\}$ showed in Lemma 3.2 is of vital importance for the convergence proof. Thus, we call $H(x,y)$ the *key function*.

More precisely, we have the following lemma.

#### Lemma 3.2

*Let* $H(z,w)$ *be defined as in* (11). *Then under assumptions* (H1)-(H6), *we have*

12 $$ H(z_{k+1},z_{k})+m \Vert z_{k+1}-z_{k} \Vert ^{2}\leq H(z_{k},z_{k-1}), $$

*where* $z_{k}=(x_{k},y_{k})$, *that is*, *the sequence* $\{H(z_{k+1},z_{k})\}$ *is decreasing*.

#### Proof

Set $m:=m_{2}-m_{1}>0$. Then the result follows directly from (9) or (10). □

### Norm estimate of the subdifferential of *H*

In this subsection, we prove that there exists a subgradient lower bound for the iterate gap. First, we estimate the norm of the subdifferential of *L*.

#### Lemma 3.3

*Define*

13 $$\begin{aligned} & p_{k+1}:=\nabla_{x}R(x_{k+1},y_{k+1})- \nabla_{x}R(x_{k+1},y_{k}) \\ &\phantom{p_{k+1}:=}{}-\frac {1}{\lambda_{k}}(x_{k+1}-x_{k})- \alpha_{k}(x_{k}-x_{k-1}), \end{aligned}$$

14 $$\begin{aligned} & q_{k+1}:=-\frac{1}{\mu_{k}}(y_{k+1}-y_{k})+ \beta_{k}(y_{k}-y_{k-1}). \end{aligned}$$

*Then*, *under assumptions* (H1)-(H6), $(p_{k+1},q_{k+1})\in\partial L(x_{k+1},y_{k+1})$. *Moreover*, *if* $\{(x_{k},y_{k})\}$ *is bounded*, *then there exists a positive constant* $C_{1}>0$ *such that*

$$\begin{aligned} &\bigl\Vert (p_{k+1},q_{k+1}) \bigr\Vert \\ &\quad\leq C_{1} \bigl( \Vert x_{k+1}-x_{k} \Vert + \Vert x_{k}-x_{k-1} \Vert + \Vert y_{k+1}-y_{k} \Vert + \Vert y_{k}-y_{k-1} \Vert \bigr). \end{aligned}$$

#### Proof

According to the definition of $x_{k+1}$ and $y_{k+1}$ and the Fermat rule, we get

$$\begin{aligned} 0&\in \partial_{x}L(x_{k+1},y_{k})+ \frac{1}{\lambda _{k}}(x_{k+1}-x_{k})+\alpha_{k}(x_{k}-x_{k-1}) \\ &= \partial f(x_{k+1})+\nabla_{x}R(x_{k+1},y_{k})+ \frac{1}{\lambda _{k}}(x_{k+1}-x_{k})+\alpha_{k}(x_{k}-x_{k-1}), \\ 0&\in \partial_{y}L(x_{k+1},y_{k+1})+ \frac{1}{\mu _{k}}(y_{k+1}-y_{k})+\beta_{k}(y_{k}-y_{k-1}) \\ &=\nabla_{y}R(x_{k+1},y_{k+1})+\partial g(y_{k+1})+\frac{1}{\mu _{k}}(y_{k+1}-y_{k})+ \beta_{k}(y_{k}-y_{k-1}). \end{aligned}$$

Thus

$$\begin{aligned} p_{k+1}:={}&-\frac{1}{\lambda_{k}}(x_{k+1}-x_{k})-\alpha _{k}(x_{k}-x_{k-1})\\ &{}-\nabla_{x}R(x_{k+1},y_{k})+ \nabla_{x}R(x_{k+1},y_{k+1}) \\ \in{}& \partial f(x_{k+1})+\nabla_{x}R(x_{k+1},y_{k+1})= \partial _{x}L(x_{k+1},y_{k+1}) \end{aligned}$$

and

$$\begin{aligned} q_{k+1}&:=-\frac{1}{\mu_{k}}(y_{k+1}-y_{k})- \beta_{k}(y_{k}-y_{k-1}) \\ &\in\nabla_{y}R(x_{k+1},y_{k+1})+\partial g(y_{k+1})=\partial _{y}L(x_{k+1},y_{k+1}). \end{aligned}$$

Using assumption (H3), we obtain that

$$\Vert q_{k+1} \Vert \leq\frac{1}{\mu_{k}} \Vert y_{k+1}-y_{k} \Vert +\beta_{k} \Vert y_{k}-y_{k-1} \Vert $$

and

$$\Vert p_{k+1} \Vert \leq\frac{1}{\lambda_{k}} \Vert x_{k+1}-x_{k} \Vert +\alpha _{k} \Vert x_{k}-x_{k-1} \Vert +\ell \Vert y_{k+1}-y_{k} \Vert , $$

*where* *ℓ* *is the Lipschitz constant of* $\nabla R(x,y)$ *on the bounded set* $\{(x_{k},y_{k})\}$ *.*

Hence the norm estimate can be immediately derived. □

The norm estimate of the subdifferential of *H* is a direct consequence of Lemma 3.3.

#### Lemma 3.4

*For all* $k\in\mathbb {N}$, $H(z,w)$ *has a subdifferential at* $(z_{k+1},z_{k})$ *of the form*

15 $$ \omega_{k+1}:=\left ( \begin{matrix} p_{k+1}+2m_{1}(x_{k+1}-x_{k})\\ q_{k+1}+2m_{1}(y_{k+1}-y_{k})\\ -2m_{1}(x_{k+1}-x_{k})\\ -2m_{1}(y_{k+1}-y_{k}) \end{matrix} \right ). $$

*Moreover*, *there exists a positive constant* $C_{2}>0$ *such that*

16 $$ \Vert \omega_{k+1} \Vert \leq C_{2} \bigl( \Vert z_{k+1}-z_{k} \Vert + \Vert z_{k}-z_{k-1} \Vert \bigr). $$

#### Proof

According to the definition of $H(z,w)$, we get

17 $$ \partial H(z,w)=\left ( \begin{matrix} \partial L(x,y)+ \left({\scriptsize\begin{matrix}{} 2m_{1}(x-u)\cr 2m_{1}(y-v)\end{matrix}} \right) \\ 2m_{1}(u-x) \\ 2m_{1}(v-y) \end{matrix} \right ). $$

The rest is immediately obtained. □

The norm estimate, together with the closeness of the limiting subdifferential, is used to obtain the following convergence of the subsequence of $\{x_{k},y_{k}\}$.

#### Lemma 3.5

Preconvergence result

*Under assumptions* (H1)-(H6), *we have the following statements*:

(i) $\sum_{k=1}^{\infty} \Vert z_{k+1}-z_{k} \Vert ^{2}<\infty$; *particularly*, $\Vert x_{k+1}-x_{k} \Vert \to0, \Vert y_{k+1}-y_{k} \Vert \to0, k\to\infty$;
(ii) *the sequence* $\{L(x_{k},y_{k})\}$ *is convergent*;
(iii) *the sequence* $\{H(z_{k+1},z_{k})\}$ *is convergent*;
(iv) *if* $\{(x_{k},y_{k})\}$ *has a cluster point* $(x^{*},y^{*})$, *then* $(x^{*},y^{*})\in\operatorname{crit} L$.

#### Proof

Set $a_{k}:=L(x_{k},y_{k})+m_{1}( \Vert x_{k}-x_{k-1} \Vert ^{2}+ \Vert y_{k}-y_{k-1} \Vert ^{2})$ and $b_{k}=(m_{2}-m_{1})( \Vert x_{k+1}-x_{k} \Vert ^{2}+ \Vert y_{k+1}-y_{k} \Vert ^{2})$. Then Lemma 3.2 gives $a_{k+1}+b_{k}\leq a_{k}$. Then assumption (H4) ensures that $a_{n}$ is bounded below. Thus Lemma 2.2 implies (i) and (ii). Moreover, the definition of $H(z,w)$ yields that

$$H(z_{k+1},z_{k})=L(x_{k},y_{k})+m_{1} \bigl( \Vert x_{k+1}-x_{k} \Vert ^{2}+ \Vert y_{k+1}-y_{k} \Vert ^{2} \bigr). $$

Thus (iii) is derived from (i) and (ii).

Now let $\{(x_{k_{j}},y_{k_{j}})\}$ be a subsequence of $\{(x_{k},y_{k})\}$ such that $\{(x_{k_{j}},y_{k_{j}})\}\to(x^{*},y^{*}), j\to\infty$. Since *f* is lower semicontinuous, we have

$$\liminf_{j\to\infty}f(x_{k_{j}})\geq f \bigl(x^{*} \bigr). $$

On the other hand, the definition of $x_{k+1}$ shows that

$$\begin{aligned} &f(x_{k+1})+R(x_{k+1},y_{k})+g(y_{k})+ \frac{1}{2\lambda _{k}} \Vert x_{k+1}-x_{k} \Vert ^{2}+\alpha_{k}\langle x_{k+1},x_{k}-x_{k-1} \rangle \\ &\quad\leq f \bigl(x^{*} \bigr)+R \bigl(x^{*},y_{k} \bigr)+g(y_{k})+ \frac{1}{2\lambda _{k}} \bigl\Vert x^{*}-x_{k} \bigr\Vert ^{2}+ \alpha_{k} \bigl\langle x^{*},x_{k}-x_{k-1} \bigr\rangle , \end{aligned}$$

from which we get

$$\begin{aligned} &R(x_{k+1},y_{k})-R \bigl(x^{*},y_{k} \bigr)+ \frac{1}{2\lambda _{k}} \bigl[ \Vert x_{k+1}-x_{k} \Vert ^{2}- \bigl\Vert x^{*}-x_{k} \bigr\Vert ^{2} \bigr] \\ &\qquad {}+\alpha_{k}\bigl\langle x_{k+1}-x^{*},x_{k}-x_{k-1}\bigr\rangle +f(x_{k+1}) \\ &\quad \leq f \bigl(x^{*} \bigr). \end{aligned}$$

Hence

$$\begin{aligned} &R(x_{k_{j}+1},y_{k_{j}})-R \bigl(x^{*},y_{k_{j}} \bigr)+ \frac {1}{2\lambda_{+}} \bigl[ \Vert x_{k_{j}+1}-x_{k_{j}} \Vert ^{2}- \bigl\Vert x^{*}-x_{k_{j}} \bigr\Vert ^{2} \bigr] \\ &\qquad{}+\alpha_{k_{j}} \bigl\langle x_{k_{j}+1}-x^{*},x_{k_{j}}-x_{k_{j}-1} \bigr\rangle +f(x_{k_{j}+1}) \\ &\quad \leq f \bigl(x^{*} \bigr), \end{aligned}$$

where we have used assumption (H5) and replaced $x_{k}, y_{k}$ by $x_{k_{j}}, y_{k_{j}}$.

Due to the fact that $\Vert x_{k+1}-x_{k} \Vert \to0$ from (i), we have $\Vert x_{k_{j}+1}-x_{k_{j}} \Vert \to0$. This, together with $\Vert x_{k_{j}}-x^{*} \Vert \to 0$, yields $\Vert x_{k_{j}+1}-x^{*} \Vert \to0$. Using the continuity of $R(x,y)$ by assumption (H2), the last inequality yields

$$\limsup_{j\to\infty}f(x_{k_{j}})\leq f \bigl(x^{*} \bigr). $$

Therefore

$$\lim_{j\to\infty}f(x_{k_{j}})=f \bigl(x^{*} \bigr). $$

In a similar way, we can prove that $\lim_{j\to\infty}g(y_{k_{j}})=g(y^{*})$. Combining with the continuity of $R(x,y)$, we immediately obtain that

$$L(x_{k_{j}},y_{k_{j}})\to L \bigl(x^{*},y^{*} \bigr),\quad j\to\infty. $$

On the other hand, Lemma 3.5(i) and Lemma 3.3 give $(p_{k_{j}+1},q_{k_{j}+1})\in\partial L(x_{k_{j}+1},y_{k_{j}+1})$ and $(p_{k_{j}+1},q_{k_{j}+1})\to0, j\to\infty$. Thus the closeness of the limiting subdifferential (see (6)) indicates that $0\in \partial L(x^{*},y^{*})$. □

### Analytic property of the key function *H*

Denote by Ω the set of the cluster points of the sequence $\{ (z_{k+1},z_{k})\}$.

#### Lemma 3.6

*Suppose that the sequence* $\{(x_{k},y_{k})\}$ *is bounded*. *Under assumptions* (H1)-(H6), *we have that*

(i) Ω *is nonempty*, *compact*, *and connected*. *Moreover*, $\operatorname{dist}((z_{k+1},z_{k}),\Omega)\to0$ *as* $k\to\infty$.
(ii) $\Omega\subset\operatorname{crit} H=\{(z^{*},z^{*}):z^{*}=(x^{*},y^{*})\in\operatorname{crit} L\}$.
(iii) *H* *is finite and constant on* Ω.

#### Proof

(i) It is easy to check by some elementary tools (see, e.g., [16]).

(ii) According to Lemma 3.5(i), $\sum_{k=1}^{\infty} \Vert z_{k+1}-z_{k} \Vert ^{2}<\infty$, and hence $\Vert z_{k+1}-z_{k} \Vert \to0, k\to \infty$. Note that $z_{k}=(x_{k},y_{k})$, so $\Omega\subset\{ (z^{*},z^{*}):z^{*}=(x^{*},y^{*})\in\operatorname{crit} L\}$. On the other hand, from (15) we see that

$$0\in\partial H(z,w)\quad\Leftrightarrow\quad u=x,v=y \quad\text{and}\quad 0\in\partial L(x,y). $$

Thus $\operatorname{crit} H=\{(z^{*},z^{*}):z^{*}=(x^{*},y^{*})\in\operatorname{crit} L\}$, and hence $\Omega\subset\operatorname{crit} H$.

(iii) Notice that $\{L(x_{k},y_{k})\}$ is convergent by Lemma 3.5(ii). Let $L^{*}=\lim_{k\to\infty}L(x_{k},y_{k})$ be a constant. For any $(z^{*},z^{*})\in\Omega$, we have $z^{*}=(x^{*},y^{*})\in\operatorname{crit} L$, and there exits a subsequence $\{(x_{k_{j}},y_{k_{j}})\}$ of $\{(x_{k},y_{k})\}$ such that $\{(x_{k_{j}},y_{k_{j}})\}\to(x^{*},y^{*})$. So

$$H(z_{k_{j}},z_{k_{j-1}})\to H \bigl(z^{*},z^{*} \bigr),\quad j\to\infty. $$

Thus

$$H \bigl(z^{*},z^{*} \bigr)=\lim_{j\to\infty}H(z_{k_{j}},z_{k_{j-1}})= \lim_{j\to \infty}L(x_{k_{j}},y_{k_{j}})=L^{*}. $$

 □

#### Theorem 3.1

Convergence

*Assume that* $H(z,w)$ *is a KL function and that the sequence* $\{ (x_{k},y_{k})\}$ *is bounded*. *Then*, *under assumptions* (H1)-(H6), *we have*

(i) $\sum_{k=1}^{\infty} \Vert z_{k}-z_{k-1} \Vert <\infty$, *that is*, $\sum_{k=1}^{\infty}( \Vert x_{k}-x_{k-1} \Vert + \Vert y_{k}-y_{k-1} \Vert )<\infty$;
(ii) $\{(x_{k},y_{k})\}$ *converges to a critical point* $(x^{*},y^{*})$ *of* $L(x,y)$.

#### Proof

According to Lemma 3.6, we consider an element $(x^{*},y^{*})\in\operatorname{crit} L(x,y)$ such that $(z^{*},z^{*})\in \Omega$, where $z^{*}=(x^{*},y^{*})$. From the previous proof we can easily obtain that $\lim_{k\to\infty }H(z_{k+1},z_{k})=H(z^{*},z^{*})$. Next, we prove the theorem in two cases.

*Case* 1. *There exists a positive integer* $k_{0}$ *such that* $H(z_{k_{0}+1},z_{k_{0}})=H(z^{*},z^{*})$ *.*

Since $\{H(z_{k+1},z_{k})\}$ is decreasing, we know that $H(z_{k+1},z_{k})=H(z^{*},z^{*})$ for all $k\geq k_{0}$. This, together with the definition of *H*, shows that $z_{k}=z_{k_{0}}$ for all $k\geq k_{0}$, and the desired results follow.

*Case* 2. $H(z_{k+1},z_{k})>H(z^{*},z^{*})$ *for all* $k\in\mathbb {N}$ *.*

Since *H* satisfies the KL property, Lemma 2.1 says that there exist $\epsilon, \eta>0$ and a concave function *φ* such that

(i) $\varphi(0)=0$;
(ii) *φ* is continuously differentiable on $(0,\eta)$ and continuous at 0;
(iii) $\varphi'(s)>0$ for all $s\in(0,\eta)$;
(iv) for all
  18 $$\begin{aligned} (z,w)\in{}& \bigl\{ (z,w)\in\mathbb {R}^{n}\times\mathbb {R}^{m}: \operatorname{dist} \bigl((z,w),\Omega \bigr)< \epsilon \bigr\} \\ &{}\cap \bigl\{ (z,w)\in\mathbb {R}^{n}\times\mathbb {R}^{m}: H \bigl(z^{*},z^{*} \bigr)< H(z,w)< H \bigl(z^{*},z^{*} \bigr)+\eta \bigr\} , \end{aligned}$$
  we have
  $$\varphi' \bigl(H(z,w)-H \bigl(z^{*},z^{*} \bigr) \bigr) \operatorname{dist} \bigl(0, \partial H(z,w) \bigr)\geq1. $$
  Notice that $H(z_{k+1},z_{k})\to H(z^{*},z^{*})$, $k\to\infty$, and $H(z_{k+1},z_{k})>H(z^{*},z^{*})$. Let $k_{1}$ be such that $H(z^{*},z^{*})< H(z_{k+1},z_{k})< H(z^{*},z^{*})+\eta$ for all $k\geq k_{1}$. By Lemma 3.6(i) there exists $k_{2}$ such that $\operatorname{dist}((z_{k+1},z_{k}),\Omega)<\epsilon$ for all $k\geq k_{2}$. Take $k_{3}=\max\{k_{1},k_{2}\}$. Then, for $k\geq k_{3}$, $\{(z_{k+1},z_{k})\}$ belongs to the intersection in (18). Hence
  $$\varphi' \bigl(H(z_{k+1},z_{k})-H \bigl(z^{*},z^{*} \bigr) \bigr)\operatorname{dist} \bigl(0,\partial H(z_{k+1},z_{k}) \bigr) \geq1, \quad \forall k\geq k_{3}. $$

Due to the concavity of *φ*,

$$\begin{aligned} &\varphi \bigl(H(z_{k},z_{k-1})-H \bigl(z^{*},z^{*} \bigr) \bigr)- \varphi \bigl(H(z_{k+1},z_{k})-H \bigl(z^{*},z^{*} \bigr) \bigr) \\ &\quad\geq\varphi ' \bigl(H(z_{k},z_{k-1})-H \bigl(z^{*},z^{*} \bigr) \bigr) \bigl(H(z_{k},z_{k-1})-H(z_{k+1},z_{k}) \bigr) \\ &\quad\geq\frac{H(z_{k},z_{k-1})-H(z_{k+1},z_{k})}{\operatorname{dist}(0,\partial H(z_{k},z_{k-1}))},\quad k>k_{3}. \end{aligned}$$

By Lemma 3.4 there exist a point $\omega_{k+1}\in\partial H(z_{k+1},z_{k})$ defined as in (15) and a positive constant $C_{2}>0$ such that

$$\Vert \omega_{k+1} \Vert \leq C_{2} \bigl( \Vert z_{k+1}-z_{k} \Vert + \Vert z_{k}-z_{k-1} \Vert \bigr). $$

Thus

$$\begin{aligned} &\varphi \bigl(H(z_{k},z_{k-1})-H \bigl(z^{*},z^{*} \bigr) \bigr)- \varphi \bigl(H(z_{k+1},z_{k})-H \bigl(z^{*},z^{*} \bigr) \bigr) \\ &\quad\geq\frac {H(z_{k},z_{k-1})-H(z_{k+1},z_{k})}{C_{2}( \Vert z_{k}-z_{k-1} \Vert + \Vert z_{k-1}-z_{k-2} \Vert )},\quad k>k_{3}. \end{aligned}$$

From Lemma 3.2 we have $H(z_{k},z_{k-1})-H(z_{k+1},z_{k})\geq m \Vert z_{k+1}-z_{k} \Vert ^{2}$. Thus

19 $$\begin{aligned} &\varphi \bigl(H(z_{k},z_{k-1})-H \bigl(z^{*},z^{*} \bigr) \bigr)-\varphi \bigl(H(z_{k+1},z_{k})-H \bigl(z^{*},z^{*} \bigr) \bigr) \\ &\quad \geq\frac {m \Vert z_{k+1}-z_{k} \Vert ^{2}}{C_{2}( \Vert z_{k}-z_{k-1} \Vert + \Vert z_{k-1}-z_{k-2} \Vert )},\quad k>k_{3}. \end{aligned}$$

Set $b_{k}=\frac{C_{2}}{m}(\varphi(H(z_{k},z_{k-1})-H(z^{*},z^{*}))-\varphi (H(z_{k+1},z_{k})-H(z^{*},z^{*})))\geq0$, $a_{k}= \Vert z_{k}-z_{k-1} \Vert \geq0$. Then (19) can be equivalently rewritten as

20 $$ b_{k}\geq\frac{a_{k+1}^{2}}{a_{k}+a_{k-1}},\quad k>k_{3}. $$

Since $\varphi\geq0$, we know that

$$\sum_{k=1}^{N}b_{k}\leq \frac{C_{2}}{m}\varphi \bigl(H(z_{1},z_{0})-H \bigl(z^{*},z^{*} \bigr) \bigr), \quad \forall N\in\mathbb {N}, $$

and hence $\sum_{k=1}^{\infty}b_{k}<\infty$. Note that from (20) we have

$$a_{k+1}\leq\sqrt{b_{k}(a_{k}+a_{k-1})}\leq \frac{1}{4}(a_{k}+a_{k-1})+b_{k},\quad k>k_{3}. $$

So Lemma 2.3 gives that $\sum_{k=1}^{\infty}a_{k}<\infty$, that is, $\sum_{k=1}^{\infty} \Vert z_{k}-z_{k-1} \Vert <\infty$, which is equivalent to $\sum_{k=1}^{\infty}( \Vert x_{k}-x_{k-1} \Vert + \Vert y_{k}-y_{k-1} \Vert )<\infty$. This indicates that $\{z_{k}\}$ is a Cauchy sequence. So $\{z_{k}\}=\{(x_{k},y_{k})\}$ is convergent. Let $(x_{k},y_{k})\to(x^{*},y^{*}), k\to\infty$. According to Lemma 3.5(iv), it is clear that $(x^{*},y^{*})$ is a critical point of *H*. □

## Conclusion

In this paper, we present a proximal alternating minimization algorithm with inertial effect for the minimization problem of the type $L(x,y)=f(x)+R(x,y)+g(y)$, where *f* and *g* are both nonconvex nonsmooth functions, and *R* is a smooth function. We prove that every bounded sequence generated by the algorithm converges to a critical point of *L*. The key point is to construct a function *H* (see (11)) that satisfies the Kurdyka-Lojasiewicz inequality. It is worth mentioning that assumption (H6) requires

$$\max\{\alpha,\beta\}\cdot\max\{\lambda_{+},\mu_{+}\}< \frac{1}{2}, $$

which can be achieved by appropriate choice of the parameters.
